# Cytogenomic features of Richter transformation

**DOI:** 10.1186/s13039-023-00662-0

**Published:** 2023-11-08

**Authors:** Renata Woroniecka, Grzegorz Rymkiewicz, Zbigniew Bystydzienski, Barbara Pienkowska-Grela, Jolanta Rygier, Natalia Malawska, Katarzyna Wojtkowska, Nikolina Goral, Katarzyna Blachnio, Marcin Chmielewski, Magdalena Bartnik-Glaska, Beata Grygalewicz

**Affiliations:** 1 Cytogenetic Laboratory, Maria Sklodowska-Curie National Research Institute of Oncology, 5 Roentgen Street, Warsaw, Poland; 2 Flow Cytometry Laboratory, Department of Cancer Pathomorphology, Maria Sklodowska - Curie National Research Institute of Oncology, Warsaw, Poland; 3 Department of Medical Genetics, Institute of Mother and Child, 01-211 Warsaw, Poland

**Keywords:** Chronic lymphocytic leukemia, Small lymphocytic lymphoma, Richter transformation, High-grade B-cell lymphoma with 11q aberrations, 11q gain/loss, IGH rearrangement, IGH deletion, MYC, CDKN2A, TP53, 13q14 deletion

## Abstract

**Background:**

Richter transformation (RT) is the development of aggressive lymphoma in patients with chronic lymphocytic leukemia (CLL) or small lymphocytic lymphoma (SLL). This rare disease is characterised by dismal prognosis. In recent years, there has been a deeper understanding of RT molecular pathogenesis, and disruptions of apoptosis (*TP53*) and proliferation (*CDKN2A, MYC, NOTCH1*) has been described as typical aberrations in RT.

**Results:**

A single-institution cohort of 33 RT patients were investigated by karyotyping, fluorescence in situ hybridization and single nucleotide polymorphism/copy number (CN) arrays. Most of RTs were typically manifested by diffuse large B-cell lymphoma, not otherwise specified, among the remaining cases one was classified as high-grade B-cell lymphoma with 11q aberrations. The most frequent alterations (40–60% of cases) were represented by *MYC* rearrangement/gain, deletions of *TP53* and *CDKN2A*, *IGH* rearrangement and 13q14 deletion. Several other frequent lesions included losses of 14q24.1-q32.33, 7q31.33-q36.3, and gain of 5q35.2. Analysis of 13 CLL/SLL-RT pairs showed that RT arised from the CLL/SLL by acquiring of 10 ~ 12 cytogenetic or CN lesions/case, but without acquisition of loss of heterozygosity regions. Our result affirmed the higher genetic complexity in RT than CLL/SLL and confirmed the linear features of RT clonal evolution as predominant.

**Conclusions:**

Cytogenomic profile was concordant with the literature data, however the role of *IGH* rearrangement, 14q deletion and 5q35.2 gain need to be explored. We anticipate that further characterization of RT lesions will probably facilitate better understanding of the RT clonal evolution.

**Supplementary Information:**

The online version contains supplementary material available at 10.1186/s13039-023-00662-0.

## Introduction

Richter transformation (RT) (previously named Richter syndrome) is the conversion of chronic lymphocytic leukemia (CLL) or small lymphocytic lymphoma (SLL) into aggressive B-cell lymphoma [1, 2]. This transformation occurs in 2–10% of CLL/SLL patients most often as diffuse large B-cell lymphoma, not otherwise specified (DLBCL, NOS), and less frequently as Hodgkin lymphoma (HL) [3–5]. The median time between CLL/SLL diagnosis and RT transformation is ~ 2 years and RT may occur in previously untreated patients [6]. The risk of RT most likely relates to underlying disease biology, rather than treatment received. Among numerous randomized trials in the front-line and relapsed/refractory settings, no significant difference in RT incidence between treatment arms were demonstrated [7–9]. Based on the analysis of immunoglobulin genes, most of the RT-DLBCL,NOS (~ 80%) are clonally related to the preceding CLL phase, thus representing true transformations [8, 10].

The clinical course of RT is poor, with median overall survival 1–2 years. However, significant difference is observed when considering the clonal relatedness to the CLL phase. Indeed, clonally related cases demonstrate a median survival of 8–16 months, while clonally unrelated RT cases show a median survival ~ 5 years [11]. Despite the huge progress in the treatment of CLL in recent years, RT also develops in patients treated with novel agents (Bruton’s tyrosine kinase and BCL2 inhibitors), and still constitutes an important clinical problem.

RT is characterised by higher molecular complexity than CLL and commonly involve lesions of suppression regulators (deletion and/or mutation of *TP53*), cell cycle (loss of *CDKN2A*), and cell proliferation (*NOTCH1* mutation, *MYC* activation) [11].

Here we present karyotype, fluorescence in situ hybridization (FISH) and microarray analysis of 33 RT patients. Combination of these three methods in relatively large group of RT patients might yield a more comprehensive profile of cytogenomic alterations in this disease—due to low incidence rate of Richter transformation, current understanding of RT genetic features is still developing.

## Materials and methods

### Patients

The study group included patients diagnosed at Maria Sklodowska-Curie National Research Institute of Oncology, Warsaw, Poland from 2004 to 2023. At that time, 49 patients who developed RT were identified. Among these, there were 5 patients with RT-HL. Thirty three RT patients were included in this study. The basis for inclusion was availability of material for genetic investigation. Due to low percentage of neoplastic cells, we did not incorporate patients with RT-HL diagnosis. Patient’s samples were analysed by flow cytometry (FCM), cytological smear, chromosome banding analysis and FISH. Some RT cases had histopathological and immunohistochemical examination (Table 1). The flow cytometric and cytogenetic diagnosis of CLL has been based mainly on peripheral blood (PB) or bone marrow (BM), while the diagnosis of SLL cases has been established mostly on the basis of fine needle aspiration biopsy (FNAB). The RT diagnostics algorithm based on FCM was previously published, and according to it, most of the material for FCM and cytogenetics has been collected by FNAB of lymph nodes (LN) or extranodal tumours [12]. In thirteen RT patients the sequential samples of paired CLL/SLL phase were examined. Array analysis was performed in ten patients.The cytogenetic data of six patients were included in the previous publication [12]. The molecular assessment of the clonal relationship between RT and the underlying CLL/SLL was not performed. However, taking into account the similarity of CLL/SLL and RT immunophenotypes and the similarity of the light and heavy chains expression, we use the FCM algorithm to estimate RT clonality [12].

**Table 1:** Demographic, diagnostic data and karyotypes of Richter transformation patients

Patient	Gender/age (years); clinical presentation at RT diagnosis; survival (months)	RT diagnosis using FNAB (Yes/No); diagnostic material	RT diagnosis using HP/IHC (Yes/No), diagnostic material	RT diagnosis – type of neoplasm (clonality)	Karyotype
1^a^	F/63; SLL; D(1)	Yes; LNc	No	DLBCL, NOS (r)	42~46,XX,der(1)(1pter→?1q32::?1q32→?1q21::?1q32→?1q21:)[8],-2[3],add(3)(q21)[8], -4[3],add(7)(q3?6)[4],-9[8],add(12)(p1?2)[7],+14 or +15 [6],-17[4],-18[4],-19[5],-21[5],+3~4mar[cp8]/46,XX[4]
2	F/62; SLL; D(3)	Yes; LNc	Yes, LNc	DLBCL, NOS (r)	44~48,XX,-1,add(6)(q1?5),del(7)(q32),+del(7)(q32),-9,del(12)(q13),add(17)(p11),-21,der(?)t(?;1)(?;p13), der(?;?)(?→cen→?::?→cen→?::12q13→12qter),+3~4mar[cp14]
3^b^	M/28; SLL; D(4)	Yes; LNc	Yes, LN	DLBCL, NOS (r)	46,XY,del(2)(q23 or q31)[7]/47,sl,+3[2]/48,sdl1,+8[8]/46,XY[4]
4^a,b^	M/67; SLL; D(3)	Yes; LNc	No	DLBCL, NOS (r)	38~47,XY,add(1)(p3?4)[7],-9[12],der(9)del(9)(p2?)del(9)(q1?3)[12],add(9)(q?13)[20],del(11)(q?21q?23)[14],-17[5],+18[3],-21[8],+22[3],+der(?;?)(?→cen→?::?→cen→?::3q13→3qter)[24][cp26]
5	M/55; CLL; D(<1)	No; PB	No	B-LBL (u)	46~47,XY,add(14)(q32),+add(18)(q2?),+mar[cp7]/46,XY[4]
6	F/62; SLL; D(19)	Yes; LNc, PB	Yes, BM	DLBCL, NOS (r)	53~59,<2n>,XX,+?del(1)(q2?1)[2],+3[9],+4[6],+5[10],+del(6)(q13)[8],+7 [8],+del(7)(q22)[2],+del(9)(q2?2)[2],+13[7],15[4],+16[8],+del(17)(p11)[6],+18[3],+19[8],+der(?19)t(1;?19)(p13;q21)[3],+20[11],+21[4],+21[6],+mar[cp12]
7^a^	F/51; CLL; D(1)	No; PB	Yes, LI	DLBCL, NOS (r)	45,XX,der(17)t(17;18)(p11.2;q11.2),-18[11]/45,idem,t(7;8)(p13;q24)[8]/46,XX[6]
8^a,b^	F/63; CLL; nd	No; PB	No	DLBCL, NOS (r)	nd
9	M/62, SLL D(6)	Yes; ST	Yes, ST	DLBCL, NOS (u)	48,XY,add(8)(p?11.2),der(14)t(8;14)(q24;q32),+2mar[7]/48,idem,del(12)(p1?3)[3]
10	F/75; SLL; D(3)	Yes; LNc	No	DLBCL, NOS (r)	42~44,X,-X,-17,+mar1,inc[cp2]
11	F/83; SLL; D(5)	Yes; LNinq	No	PBL (u)	45~47,-X[6],add(X)(p22)[4],-2[3],del(6)(q21)[6],?add(14)(q32)[3],-16[5],der(19)t(11;19)(q13;p13.3)[5],-22[4],+1~7mar[cp7]/46,XX[1]
12^a,b^	M/62; SLL; D(7)	Yes; LNc, PB	No	DLBCL, NOS (r)	71~86,XX,-Y,-1,add(3)(q2?7)x2,-4,-4,-5,-6,add(7)(q32)x3,-8,add(10)(q26)x2,add(11)(q23)x2,-12,-12,-13,-13,add(13)(q3?4)x2,-14,-14,add(14)(q32)x2,add(15)(p11)x2,-16,-17,+18,der(?)t(?;12) (?;q15)x2,+1~4mar[cp8]
13^b^	M/81; SLL; D(1)	Yes; LNc	No	DLBCL, NOS (r)	46,XY,-13,add(14)(q32),+mar[4]/46,XY[5]
14	F/67; SLL; nd	Yes; LNc, PB	Yes, LN, BM	DLBCL, NOS (r)	44~47,XX,-1[8],-4[3],add(7)(q22)[4],-13[6],-14[8], +der(14)(?::14q32→14p11.2::?→ cen→?::14p11.2→14q32::?)[9],-15[5],-16[7],-18[5],add(19)(p13)[5],+der(?)t(1;?)(p13;?)[9], +4~7mar[cp9]/ 46,XY[3]
15^a^	M/72; SLL; D(2)	Yes; LNab	No	DLBCL, NOS (u)	47~50,XY,add(1)(q21)[10],+del(1)(p?31)[10],-2[6],del(3)(p12p14)[9], add(4)(q35)[10],-5[9],-7[10],-9[9],add(11)(q21)[7],-12[3],-13[7],-16[8],i(17)(q10)[10],-18[9],+20[10],+20[8], +21[5],+6-12mar[cp10]
16	F/70; SLL; D(11)	Yes, LNc, SofT	Yes, LN, SofT	DLBCL, NOS (u)	44~44,XX,del(1)(p22p32),+del(1)(q3?2),add(8)(q24),-9,-11,add(13)(p13),-16,-17,-18, +7~11mar[cp4]
17^a,b^	M/65; SLL; A(60)	Yes; LNc	No	HGBL-11q (u)	47,X,-Y,dup(11)(q22q23),+12,+mar1[4]/47,idem,add(18)(q23)[5]
18.^b^	F/62; SLL; D(6)	Yes; LNc	No	DLBCL, NOS (r)	46,XX,add(1)(q21),+del(1)(p22),-4,del(6)(q2?3),ins(7;?)(q22;?),-10,der(11)t(1;11)(q21;p15),del(13)(q?14q?22),add(19)(q13), +mar1[10]
19^a,b^	F/50; CLL; D(2)	Yes; LNc, PB	Yes, BM	DLBCL, NOS (r)	46~49,XX,del(1)(p13),+del(1)(q12),-2, add(7)(q36),+add(7)(q36),t(8;14)(q24;q32),-9, add(10)(p13),der(14)t(9;14)(q13;p11.2),del(15)(q2?4),i(17)(q10),add(19)(q13),-22, add(22)(q11),+1~2mar[cp20]
20^a^	M/71; SLL; D(2)	Yes; LNc	Yes, LN	HS (u)	47,XY,+i(12)(q10),?t(15;22)(q15;q13)[9]/46,XY[2]
21^b^	F/66; CLL; D(7)	Yes; LN, PB,	Yes, BM	DLBCL, NOS (r)	49,X,-X,add(1)(p13),t(2;?20)(p11.2;p11.2),+3,+6,del(6)(q2?3), t(8;14)(q24;q32),+18,+mar1[10]
22	M/60; SLL; D(4)	Yes; LNc	Yes, LNc	DLBCL, NOS (u)	45,XY,+X,del(1)(p32),-2,-7,del(11)(q21),-14,add(15)(p13),add(16)(q24),del(17)(p11),+mar1[7]/46,XY[1]
23^a,b^	M/42; CLL; D(2)	No; PB, BM	Yes, BM	DLBCL, NOS (r)	42~43,X,-Y,der(1)add(1)(p36)del(1)(q25)[5],-3[3],-4[5],add(4)(q3?5)[5],-5[3],add(7)(q36)[4],-8[5],-9[3],-10[4],-12[5],-13[5],add(15)(q15)[5], +mar1[3],+mar2[5],+mar3[4][cp5]/ 43~45,XY,add(1)(q4?4)[4],-4[4],add(4)(q3?5)[4],add(7)(q36)[4],-8[4],-9[4],-10[3],-12[4],-13[4], add(15)(q15)[4],+mar1[4],+mar2[2],+3mar[3][cp4]/ 45,XY[1]
24.	F/68; CLL; D(8)	No; BM	Yes, BM	DLBCL, NOS (r)	54,X,der(X)t(X;1)(p22;p13),-1,del(4)(q2?6),add(8)(p?11.2),+9,del(10)(p12),+11,+13,+13, del(13)(q2?1q2?2),+14,+19,-20,+21,+der(?)t(?;1)(?;q21),+mar1,+mar2[8]/46,XX[3]
25	F/61; CLL; D(<1)	Yes; LNc, PB	No	DLBCL, NOS (r)	46,XX,add(1)(p36),-5,ins(7;?)(q22;?),-8,del(9)(q?21q?22),-16,del(17)(p11.2),add(19)(q13),+mar1,+mar2,+mar3[10]
26^a^	M/63; CLL; A(68)	Yes; LNc,	Yes, LN	DLBCL, NOS (u)	46~50,XY,+X[3],add(1)(p36)[6],-4[5],+7[5],+10[4],+11[6],t(14;18)(q32;q21)[5],add(15)(q26)[5],+mar1[5],+mar2[3],+1~4mar[4][cp6]/46,XY,add(6)(q2?7)[2]/46,XY[2]
27	M/81; SLL; D(1)	Yes; LNc,	No	DLBCL, NOS (r)	46,XY,-13,add(14)(q32),+mar[4]/46,XY[5]
28^a^	M/53; SLL; D(4)	Yes; jaw tumor, PF	Yes, oral gingiva	DLBCL, NOS (u)	44~47,XX,del(1)(p2?2)[2],del(1)(q12)[2],+7[3],del(8)(q24)[2],add(14)(q32)[3],-17[3],+der(?)t(?;1)(?;q21)[3][cp4]/46,XX[1]
29	F/94; CLL; A(5)	Yes; LNax, PB	No	DLBCL, NOS (u)	46,XX,del(1)(p13),der(1)add(1)(p3?6)?add(1)(q25),+del(3)(q2?5),del(6)(q21),i(6)(p10),add(9)(p13),del(13)(q22),-14,-15,+18,add(19)(p13),-21,-22,+mar1,+mar2[10]
30^a^	M/71; SLL; D(2)	Yes; LNab	No	HGBL, NOS (u)	47~50,XY,add(1)(q21)[10],+del(1)(p?31)[10],-2[6],del(3)(p12p14)[9],add(4)(q35)[10],-5[9],-7[10],-9[9],add(11)(q21)[7],-12[3],-13[7],-16[8],i(17)(q10)[10],-18[9],+20[10],+20[8],+21[5],+6~12mar[cp10]
31	M/78; SLL; D(<1)	Yes; LNil	No	DLBCL, NOS (r)	nd
32	M/87; CLL; nd	No; PB	No	DLBCL, NOS (r)	47,XY,+5,del(2)(p11.2),der(8)t(2;8)(p11.2;q24),del(11)(q21),der(14)t(8;14)(q24;q32)[15]
33	M/66; CLL; D(9)	Yes; LNax	Yes, LN	DLBCL, NOS (r)	85~93,XXYY,+mar[4][cp6]/46,XY[2]

^a^RT cases with CLL/SLL paired data, ^b^RT cases with array examination

*A* Alive, *BM* Bone marrow/trephine bone marrow biopsy, *B*-*LBL*: B-lymphoblastic lymphoma, *CLL* Chronic lymphocytic leukemia, *D* Dead, *DLBCL,NOS: *Diffuse large B-cell lymphoma, not otherwise specified, *FNAB* Fine needle aspiration biopsy, *HP/IHC* Histopathological/immunohistochemical examination, *HGBL,NOS* High-grade B-cell lymphoma, not otherwise specified, *HGBL-11q *High-grade B-cell lymphoma with 11q aberrations, *nd* no data, *HS* Myeloid/histiocytic sarcoma, *LI *Large intestine, *LN *Lymph node, *LNab* Abdominal lymph node, *LNax* Axillary lymph node, *LNc* Cervical lymph node, *LNil* iliac lymph, *LNing* inguinal lymph node, node, *PB* Peripheral blood, *PBL* Plasmablastic lymphoma, *PF* Pleural fluid, *r* Clonally related by FCM algorithm, *RT* Richter transformation, *SLL* Small lymphocytic lymphoma, *SofT* Soft tissue, *ST *Subcutaneous tumor, *u* Clonally unrelated by FCM algorithm

### Cytogenetics

The PB cells were cultured for 72 h with DSP-30 (2 μM; TIBMolBiol, Berlin, Germany) together with IL-2 (200 U/mL; R&D Systems, Minneapolis, MN, USA) (performed in our laboratory since 2012) (Additional file 1: Table). The LN, BM and extranodal tumor cells were fixed directly, cultured for 24 h without mitogen and cultured for 72 h with DSP-30. Chromosomes were stained with Wright for G,C-banding and analysed using the MetaSystems Ikaros imaging system (Metasysytems, Altlussheim, Germany). The karyotypes were described according to the International System for Human Cytogenomic Nomenclature (ISCN 2020) [13]. Complex karyotype (CK) was defined by the presence of three or more alterations in the same clone; high complex karyotype (H-CK) described karyotype with more than five aberrations.

Interphase FISH was performed on the fixed cells using commercially available probes as follows: IGH BAP, MYC BAP, IGH/MYC/CEP8, TP53, ATM, D13S319/13q34, CEP12, CDKN2A/CEP9, CCND1, KMT2A BAP,TelVysion11q, (Vysis, Abbott Molecular, Downers, Grove, IL, USA).

### Single nucleotide polymorphism/array comparative genomic hybridization (SNP/aCGH)

DNA was extracted from fresh material or cytogenetic fixed cell suspension by QIAmp DNA Blood Mini Kit (Qiagen, Valencia, CA) according to the manufacturer’s recommendation. The reference DNA was used from two pools (male and female) from normal individuals and run as a same-sex control. For SNP/aCGH analysis CytoSureTM Haematological Cancer and SNP Array (8 × 60 k) (Oxford Gene Technology (OGT), Yarnton, Oxford OX5 1PF UK) was used. Purification of labeled products, hybridization, and post-wash of the array was carried out according to OGT’s recommendation. Array slides were scanned with Agilent’s DNA Microarray Scanner with extraction software (Agilent, Santa Clara, USA). For the analysis of array data the CytoSure Interpret software 020022 (OGT) was used.

## Results

### Patients

The patient cohort consisted of 33 RT patients (Table 1). RT diagnosis was based on a LN/extranodal tissue biopsy in 27 patients (simulatonues involvement of PB or BM was confirmed in 7 patients), and on PB/BM in 6 patients. The median age at RT diagnosis of the whole case series was 65 ± 0.5 years with female:male ratio 1:1.2. RT was represented by DLBCL,NOS in most of patients (85%), and by high-grade B-cell lymphoma with 11q aberrations (HGBL-11q), high-grade B-cell lymphoma, not otherwise specified (HGBL,NOS), plasmablastic lymphoma, myeloid/histiocytic sarcoma and B-lymphoblastic lymphoma in the remaining patients. In 29 patients with available data, the median time from CLL/SLL to RT diagnosis was 13 months (range 0–83).

### Cytogenetic lesions in RT

According to the number of chromosome lesions, 30 (97%) patients had CK (*n* = 31). Among these patients, 7 patients presented less than five aberrations and 24 patients (77%) had H-CK. An average number of karyotype aberrations/case was 13 (*n* = 31, range 2–47/case) (Table 1).

**Fig. 1:**
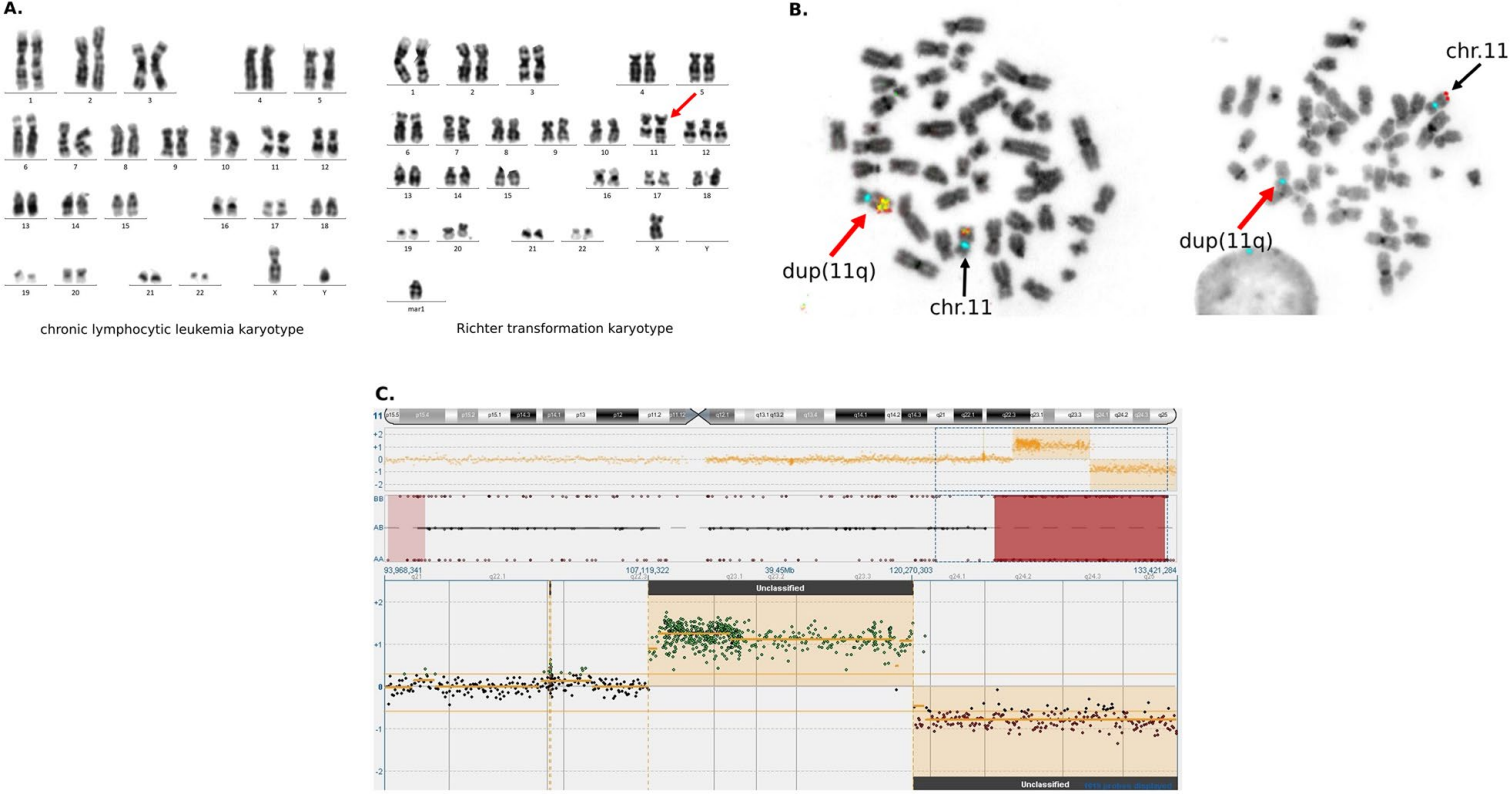
Cytogenomic data of case 17 with Richter transformation presented as high-grade B-cell lymphoma with 11q aberrations. **A** Karyotypes of RT and CLL paired phases, which demonstrate evolution of cytogenetic aberrations at RT transformation: CLL karyotype 46,XY[20], RT karyotype 47,X,-Y,dup(11)(q22q23), + 12, + mar1[4]/47,idem,add(18)(q23)[5] {red arrow indicates dup(11q)}. **B** FISH on RT metaphases (11 centromere signals are blue, red arrows indicate dup(11q) with 11q-gain/loss): a few KMT2A (yellow) signals on dup(11q) and one KMT2A signal on normal chromosome 11, 11qtel (red) signal on normal chromosome 11 and lack of 11qtel signal on dup(11q). **C** CGH/SNP array result: ideogram of chromosome 11, below copy number (CN) variations indicating duplication and deletion of 11q, underneath big brown blocks demonstrating loss of heterozygosity. Lower section shows magnification of aCGH analysis (CN variations). Green dots indicate gain, red dots shows deletion regions

In case 17 with derivative chromosome 11, FISH demonstrated the gain of *ATM* (3–5 copies) and *KMT2A* (4–6 copies), loss of tel11q and lack of *MYC* abnormality (Fig. 1). The 11q-gain/loss was confirmed by SNP/aCGH also (duplication of 13,58 MB and deletion of 14,59 MB). Taking into account cytogenomic and FCM data, the final diagnosis of HGBL-11q was established [1, 2, 14].

**Fig. 2:**
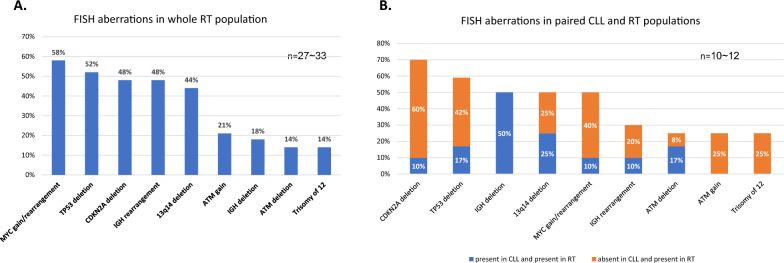
Frequency of FISH aberrations in RT patients. **A** The frequency of FISH aberrations in whole RT population. **B** The frequency of FISH aberrations in paired CLL and RT populations. Orange bars represent the frequency of FISH aberrations present in RT and absent in paired CLL, which reflect the evolution of genetic lesions during transformation. Blue bars represent the frequency of FISH aberrations present in RT and present in paired CLL, which reflect the maintaining of lesions during transformation. n—according to the availability of material, variable numbers reflect varying numbers of examined cases

The most frequent aberrations detected by FISH examination included (in descending order of frequency): *MYC* gain/rearrangement in 19 patients (58%, *n* = 33), *TP53* deletion in 16 patients (52%, *n* = 31), *CDKN2A* deletion in 16 patients (48%, *n* = 33), *IGH* rearrangement in 16 patients (48%, *n* = 33) and 13q14 deletion in 12 patients (44%, *n* = 27) (Fig. 2A). Deletion of *IGH* involved 5’ telomeric region in 3 patients and 3’ centromeric region in 3 patients. The *IGH* rearrangement led to *MYC::IGH* fusion in 5 patients, and in 3 patients *IGH* was translocated on 17p, 3q or chromosomal marker.

### SNP/aCGH lesions in RT

**Fig. 3:**
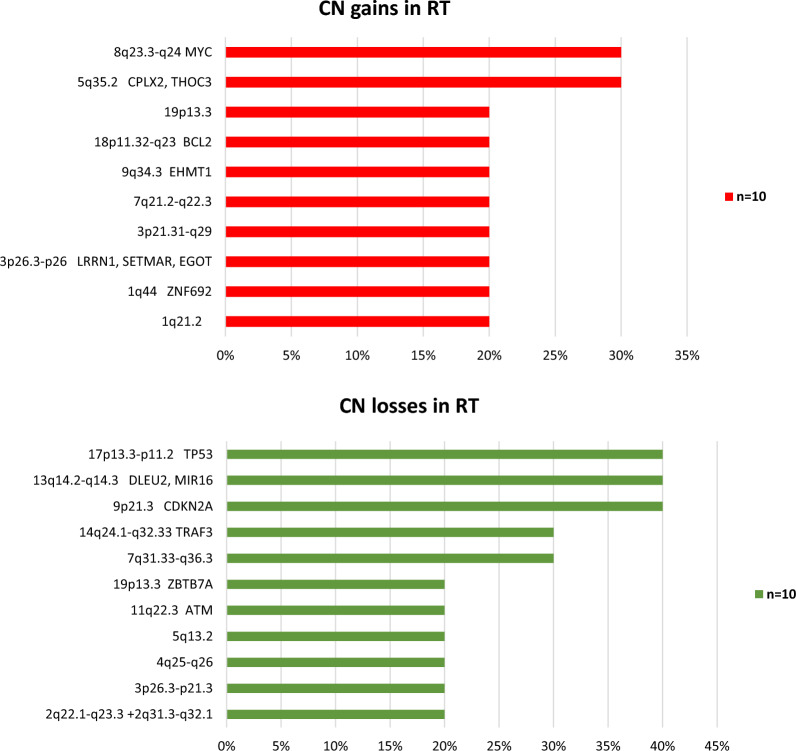
Frequency of copy number aberrations (CN) detected by arrays CGH. Red bars represent the frequency of minimal common regions (MCRs) of CN gains and green bars represent the frequency of MCRs of CN losses. The MCRs cover the genes included in the annotations

In all 10 RT patients arrays examination revealed many copy number aberrations (CNA) (Fig. 3). An average number of CNAs per case was 14 (*n* = 10, range 2–27/case). We identified 10 recurrent (observed in ≥ 2 patients) regions of loss and 11 recurrent regions of gain. Among the most frequent copy number (CN) gains were regions (in brackets: genes, which are potentially targets of the lesion): 8q23.3-q24.3 (*MYC*) and 5q35.2 (*CPLX2, THOC3*). The most frequent CN losses were regions: 17p13.3-p11.2 (*TP53*), 13q14.2-q14.3 (*DLEU, MIR16*), 9p21.3 (*CDKN2A*), 14q24.1-q32.33 (*TRAF3*) and 7q31.33-q36.3.

The loss of heterozygosity (LOH) regions were observed in 6/10 patients. An average number of LOHs per case was 3 (*n* = 10, range 0–11/case). Among recurrent (observed in ≥ 2 patients) LOHs were regions: 3p26-p25.1, 3q11.2-q12.3, 4q26-q28.3, 8q24.12-q24.13, 9p24.3-p13.2, 9q21.11-q21.13. Some of these regions (3q, 4q, 9q) were the regions of copy neutral LOH (CN-LOH), the remaining LOHs co-occurred with deletions (3p, 9p) or duplication (8q) of appropriate regions.

### Clonal evolution in transformation of CLL/SLL to RT

The assessment of the evolutionary history of CLL/SLL transformation was possible after the comparison of genetic lesions in two phases of disease. Paired genetic analysis was performed for 13 cases with samples available at both CLL/SLL and RT phase. In CLL/SLL population before RT transformation, the FISH, karyotype and CN alterations were observed sporadically. In CLL/SLL, the most frequent FISH aberrations were *IGH* deletion (50%) and 13q14 deletion (25%), followed by *TP53* and *ATM* deletions (17%) (Fig. 2B). Karyotypes of 7 CLL/SLL cases were normal or presented few aberrations with an average number of alterations ~ 1 (range 0–3). However, some limitations of CLL karyotype data should be taken into account due to the lack CpG + IL2 stimulation in several cases. Similarly as in karyotype study, an average number of CNAs in CLL/SLL was 2 (range 0–3, *n* = 4). In all patients, each FISH abnormality identified in the CLL/SLL phase was maintained in the RT phase and the transformation was characterized by acquisition of secondary aberrations, which were absent at CLL/SLL phase. The most frequently acquired FISH aberrations were *CDKN2A* deletion (60%) and *TP53* deletion (42%), followed by *MYC* alterations (40%),13q deletion (25%), *ATM* gain (25%) and trisomy 12 (25%). *IGH* deletion was the only aberration, which was not acquired during transformation, contrary to trisomy 12 and *ATM* gain, which were absent in paired CLL/SLL population. Karyotypes and CNA data of RT paired cases demonstrated higher number of aberrations when comparing to paired CLL/SLLs. An average number of karyotype lesions was 11 (range 2–16) and an average number of CNAs was 14 (range 9–20). In two RT cases karyotypes presented acquisition of unrelated lesions. In case 26, RT karyotype included unrelated clones: one with maintained SLL aberration and one with many others alterations; in case 19, lesions observed in SLL were lost at RT transformation.

Contrary to complexity of karyotype and CN aberrations, an average number of LOH was similar in RT and paired CLL/SLL populations (range 0–3, *n* = 4).

Detailed genetic data are available in Additional file 1: Table.

## Discussion

Richter transformation is rare complication of CLL/SLL with a dismal prognosis and is distinct from the progression of CLL/SLL [15]. DLBCL,NOS is the most frequent type of RT, and the minority of RT is represented by other aggressive lymphomas. In the present study, for the first time we describe HGBL-11q as the result of CLL/SLL transformation. HGBL-11q, earlier defined as Burkitt-like lymphoma with 11q aberration, is relatively new entity and the knowledge regarding this disease is still developing [1, 2]. First studies presented, that this lymphoma, which morphologically and phenotypically resembles Burkitt lymphoma (BL), has unique chromosome 11q aberrations (11q gain/loss) instead of *MYC* rearrangement [16, 17]. Subsequent data described, that some rare cases with 11q gain/loss have simultaneously rearrangement or amplification *of MYC* and are diagnosed as BL or HGBL,NOS, or even double-hit lymphoma with *BCL2* rearrangement [18–21]. All these reports showed, that HGBL-11q is primary disease, however, as we present in our study, it can also be the result of clonally unrelated CLL/SLL transformation.

In recent years, there has been a deeper understanding of RT evolution from CLL. Generally, the main problem with neoplasia evolution investigations is that they are based on the genetic analysis of single time-point samples. In this context, RT evolution studies have the advantage due to availability of paired CLL-RT samples. According to current data, in vast majority of RT cases the transformation occurs through a linear model [22]. In this type of evolution the predominant clone acquires novel genetic lesions leading to more aggressive disease [23]. For comparison, CLL progression is based on both linear and branching clonal evolution, where in branching model precursor clone diverge into separate lineages [24]. Our result confirmed, that clonal evolution of RT has predominantly features of linear model. In majority of cases, the abnormalities identified in the CLL/SLL phase were maintained in the RT phase, and RT was characterized by acquisition of new, secondary aberrations. The average numbers of karyotype and CN aberrations in CLL/SLL-phase samples were significantly lower than in RT samples, reaching in CLL/SLL phase 1 ~ 2 alterations, while in RT samples they were as high as 11 ~ 14. These results are consistent with literature data, in which RT typically arises from the predominant CLL clone by acquiring of ~ 20 genetic aberrations/case [22].

RT is characterized by high complexity of genetic alterations and by frequent lesions of *TP53*, *CDKN2A* and *MYC* [8, 22, 25]. Accordingly, almost all RT patients in our study presented the high degree of genome heterogeneity with ~ 13 karyotype aberrations or ~ 14 CNAs per case and the above aberrations were the most frequent alterations. They were observed in near half of FISH study group and in 30%-40% of microarray study group. This is consistent with published data, in which *TP53* alterations (deletions and mutations) occur in 40% to 80% of RT, deletion of *CDKN2A* occurs in ~ 30% of cases and *MYC* structural alterations are observed ~ 30% of RT [22, 26, 27]. Disruption of *TP53*, one of the most prominent tumour suppressor genes, explains the chemorefractory phenotype of RT; on the other hand, cell-cycle deregulation by *CDKN2A* deletion and *MYC* deregulation may result in the progressive biology of RT [8]. Important role of the *MYC* activation in RT pathogenesis is confirmed not only by high frequency of *MYC* alterations in RT, but also by two mutually exclusive ways of *MYC* stimulation in RT: translocations/gains of *MYC* or *NOTCH1* mutations [28]. In our study, *CDKN2A* deletion and *MYC* alterations were generally acquired at the time of transformation, what is consistent with current knowledge [8, 27]. On the other hand, deletion of *TP53* was observed both before and during transformation, similar to other studies [26, 29].

Data regarding the role of *IGH* rearrangement in biology of RT are scarce, nevertheless *IGH* appears to be target of recurrent lesion in RT. Published data showed, that *IGH* translocation leading to activation of oncogenes, is one of the most frequent aberration at clonal evolution in CLL, has intermediate-adverse prognostic impact in CLL and a distinct mutational profile from other classic cytogenetic CLL subgroups [30, 31]. In our study and previously published report, the frequency of *IGH* rearrangement in RT was remarkably high and present before and after transformation [12]. We observed that this aberration led to *MYC::IGH* fusion in some cases, and to fusions with unrecognized partners in the remaining cases.

Deletion of 13q14 is described as infrequent, but recurrent lesion in RT [22, 32]. This aberration is known to be an early event in CLL and it is not surprising to find this alteration maintained from CLL phase. According to current data, this alteration has never been acquired at RT [22]. In contrast to these data, in our report we demonstrate, that incidence of 13q14 deletion in RT was quite high and this alteration was observed not only at CLL/SLL phase, but it was also acquired during transformation also.

There are limited published literature regarding deletions of 14q in RT. Based on these data, 14q deletions are mapped between 14q23.2-q32.33 and *TRAF3* is considered as the putative target of these lesions [22]. Similarly, we observed, that 14q deletions were heterogenous in size, they covered centromeric or telomeric regions of *IGH* and, in some cases, *TRAF3*. It might be interesting to investigate this lesion in other CLL-RT populations, considering, that *IGH* deletions were the only alterations in our RT patients, which have never been acquired at transformation, but maintained from CLL/SLL phase. This feature of *IGH* deletion suggests their primary character. Wlodarska et al. described, that 5’IGH deletions reflect physiological event, however, Quintero-Rivera et al. considered these lesions as important early events in the pathogenesis of CLL [33, 34]. Quintero-Rivera F et al. based their thesis on high frequency of 5’*IGH* deletions in CLL patients, presence of these deletions in BM derived cells not yet exposed to antigen, and lack of these deletions in normal cases.

In contrary to above alterations, gain of 5q35.2 in RT has not been describe to date. This region encompass *THOC3*, which belongs to genes coding RNA-binding proteins (RBP). These genes play an important role in post-transcriptional regulation and the activity of RBP-RNA networks has been shown to be closely related to tumour development [35].

To date, the molecular profile of RT-DLBCL,NOS has been considered not to overlap with the genetics of de novo DLBCL [14]. However, comprehensive genetic analysis of primary DLBCLs by Chapuy et al. allowed to identify five robust DLBCL subsets, including subset “cluster 2” with genetic profile resembling profile of RT in our study [36]. This subset was characterised by frequent CN aberrations and harboured losses of *TP53*, *CDKN2A*, 13q14, 14q32.31 together with gains of 8q24.22, 5q,11q. Such a genetic signature contrasted with genetic profiles of remaining subsets, which were characterised mainly by mutational drivers.

Single nucleotide polymorphism arrays are powerful method, which can complement cytogenetics and CGH with unique ability to find a hidden chromosomal lesions, as CN-LOHs are. According to current knowledge, regions affected by acquired CN-LOHs include suppressor genes important for cancer genesis and evolution. The frequency of CN-LOHs varies in different types of hematological malignancies, reaching ~ 80% in acute lymphoblastic leukemia or acute myeloid leukemia [37]. In CLL patients, CN-LOH frequency is of 6–7%, which is lower than other malignancies [37]. However, little is known about CN-LOHs in RT, and reported regions are different from CN-LOHs detected in our patients [25]. Interestingly, the CLL/SLL-paired RT cases in our report demonstrated, that transformation was not accompanied by acquisition of LOHs, contrary to CNAs and cytogenetic alterations.

## Conclusions

In conclusion, our report for the first time describes HGBL-11q as Richter transformation. We affirmed *MYC*, *TP53*, *CDKN2A* lesions as the most frequent alterations in RT and identified *IGH* rearrangement and 13q14 deletion as another frequent aberrations of CLL/SLL transformation. Our result confirmed, that clonal evolution of transformation had predominantly linear features and comprised acquirement of new karyotype or CN aberrations without acquisition of LOHs. Most of RT alterations were acquired at transformation, except deletion of *IGH*, which is considered primary event in pathogenesis of CLL. We anticipate that further detailed characterization of RT clonal evolution will facilitate better understanding of the RT initiation.

### Supplementary Information

**Additional file 1.** Detailed genetic data of patients.

## Data Availability

The datasets used and/or analyzed during the current study are available from the corresponding author on reasonable request.

## Abbreviations

BM: Bone marrow
CK: Complex karyotype
CLL: Chronic lymphocytic leukemia
CN: Copy number
CNA: Copy number aberrations
CN-LOH: Copy neutral loss of heterozygosity
DLBCL: Diffuse large B-cell lymphoma
DLBCL, NOS: Diffuse large B-cell lymphoma, not otherwise specified
FISH: Fluorescence in situ hybridization
H-CK: High complex karyotype
HGBL-11q: High-grade B-cell lymphoma wit 11q aberrations
HGBL, NOS: High-grade B-cell lymphoma, not otherwise specified
LN: Lymph node
LOH: Loss of heterozygosity
PB: Peripheral blood
RT: Richter transformation
SNP/aCGH: Single nucleotide polymorphism/array comparative genomic hybridization
